# Generation of Synthetic CT Images From MRI for Treatment Planning and Patient Positioning Using a 3-Channel U-Net Trained on Sagittal Images

**DOI:** 10.3389/fonc.2019.00964

**Published:** 2019-09-25

**Authors:** Dinank Gupta, Michelle Kim, Karen A. Vineberg, James M. Balter

**Affiliations:** Department of Radiation Oncology, University of Michigan, Ann Arbor, MI, United States

**Keywords:** synthetic CT, MRCT, deep learning, MRI, radiation oncology

## Abstract

A novel deep learning architecture was explored to create synthetic CT (MRCT) images that preserve soft tissue contrast necessary for support of patient positioning in Radiation therapy. A U-Net architecture was applied to learn the correspondence between input T1-weighted MRI and spatially aligned corresponding CT images. The network was trained on sagittal images, taking advantage of the left-right symmetry of the brain to increase the amount of training data for similar anatomic positions. The output CT images were divided into three channels, representing Hounsfield Unit (HU) ranges of voxels containing air, soft tissue, and bone, respectively, and simultaneously trained using a combined Mean Absolute Error (MAE) and Mean Squared Error (MSE) loss function equally weighted for each channel. Training on 9192 image pairs yielded resulting synthetic CT images on 13 test patients with MAE of 17.6+/−3.4 HU (range 14–26.5 HU) in soft tissue. Varying the amount of training data demonstrated a general decrease in MAE values with more data, with the lack of a plateau indicating that additional training data could further improve correspondence between MRCT and CT tissue intensities. Treatment plans optimized on MRCT-derived density grids using this network for 7 radiosurgical targets had doses recalculated using the corresponding CT-derived density grids, yielding a systematic mean target dose difference of 2.3% due to the lack of the immobilization mask on the MRCT images, and a standard deviation of 0.1%, indicating the consistency of this correctable difference. Alignment of MRCT and cone beam CT (CBCT) images used for patient positioning demonstrated excellent preservation of dominant soft tissue features, and alignment comparisons of treatment planning CT scans to CBCT images vs. MRCT to CBCT alignment demonstrated differences of −0.1 (σ 0.2) mm, −0.1 (σ 0.3) mm, and −0.2 (σ 0.3) mm about the left-right, anterior-posterior and cranial-caudal axes, respectively.

## Introduction

While MRI has shown significant value for Radiation Oncology treatment of intracranial tumors due to its superior soft tissue contrast and ability to map quantitative biological features such as diffusion and perfusion, it has inherent limitations in providing electron density maps necessary to support calculation of radiation dose distributions, as well as in supporting most existing clinical workflows for patient positioning that rely on alignment of treatment planning CT images with Cone Beam CT (CBCT) scans acquired at the time of patient positioning. While the former issue has been reasonably resolved by a variety of synthetic CT approaches (1–6), the latter has received little attention.

Many CBCT-CT alignment mechanisms rely on reasonably similar intensity distributions, especially those that align soft tissue features. Recent reports have demonstrated the potential of “machine learning” approaches to generate synthetic CT (“MRCT”) scans, but have shown rather large errors in intensity differences of the soft tissues of the brain. While not specifically analyzed in most of these investigations, the structural details of soft tissue features are often misrepresented, thus potentially confounding alignment with similar features displayed on CBCT image volumes. This may present challenges for precise local alignment of tissues, as the potential for local changes between simulation and treatment is enhanced due to the temporal periods associated with frameless radiosurgery techniques (7).

The objective of this investigation was to investigate whether a Neural Network could be optimized to preserve the soft tissue contrast features necessary for precision alignment of intracranial tumors. Attempts to maximize local contrast include use of a U-Net architecture trained on aligned MR and CT pairs, training on sagittal planes to increase data diversity for the same number of input patients, and separation of the CT images into three intensity regions, preserving the narrow intensity range wherein most of the soft tissue contrast falls on CT. The impact of numbers of training images is briefly explored.

## Materials and Methods

### Training Data

Under an Institutional Review Board approved protocol, 60 patients who underwent CT-based simulation for treatment of intracranial tumors further underwent an MR simulation scan while immobilized with their fixation devices. CT image volumes were all acquired using the same in-house CT simulator (Brilliance big bore, Philips Medical Systems, Andover MA) and had initial voxel sizes ranging from 0.6 by 0.6 by 1 mm to 1.17 by 1.17 by 3 mm. MR images were acquired on an in-house 3 Tesla MR Simulator (Skyra, Siemens Healthineers, Erlangen, Germany), and included a T1-weighted acquisition as the in phase images of a Dixon scan series. These images, with sampled voxel sizes of ~1 by 1.25 by 1.25 mm, were used for training.

CT image volumes were rigidly aligned to corresponding MR images using an open source package *dipy* (8) and the resulting transforms applied to the CT and resampled to match the native MR image resolution.

**Figure 1:**
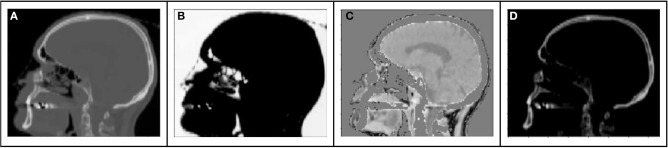
Example sagittal CT image with tissue windows: **(A)** original CT image **(B)** air window **(C)** tissue window, **(D)** bone window.

The range of Hounsfield Units of typical human tissues is roughly −1,000 to 2,000. The majority of this intensity range is occupied by air and/or skeletal tissues. Most soft tissue falls within a narrow subset of HU values (~−100 to 100 HU). As a result of this very limited region of the intensity range wherein soft tissue contrast lies, training loss functions will have HU differences an order of magnitude higher in air or bone regions than in locations consisting of primarily soft tissue contrast. As a result, the training might prioritize errors in bone or air over those of soft tissue. This leads to a potential challenge to preserving local soft tissue structures, especially with limited amounts of training data. To attempt to capture soft tissue contrast, we split training CT images into 3 separate output “channels” that can facilitate easier learning from limited data sets (Figure 1). These channels were defined by using intensity thresholds of < −100 HU to define voxels containing air, −100 HU to 100 HU to primarily identify soft tissue and >100 HU for voxels containing bone. The regions outside of the threshold masks were set to 0 HU for each channel. This 3-channel approach forces the network to learn the 3 regions of interests separately, thus capturing the tissue intensity contrasts independently for air, tissue and bone. As the tissue intensities were consistent across the MR scans due to a standard image acquisition methodology and coil configuration, and the HU value ranges of tissues were similarly consistent, a fixed normalization was applied to the input and separately each of the output channels.

### Network Architecture

**Figure 2:**
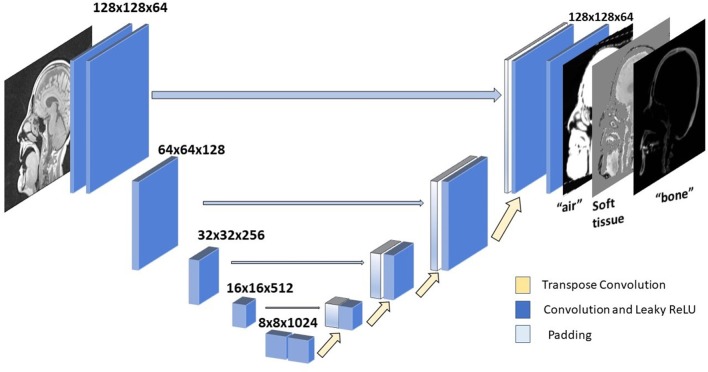
U-net architecture. Each block represents a convolution operation, followed by batch normalization and Leaky ReLU. Last convolution operation converts 64 dimensional channel to 3 channel synthetic CT.

A U-Net neural network architecture (1, 9) (Figure 2) was implemented for translating T1-weighted MRI images into corresponding MRCT images. This network involves a series of downsampling operations that squeezes the input image by factors of two while increasing the number of filters by factors of two. Once this downsampling shrinks the input image 5 times, the same number of upsampling operations successively increase the image dimension by factors of 2, while reducing the number of channels by factors of two. This upsampling is also supported by padding of weights from the corresponding dimension image in the downsampled layer. This allows for easy flow of gradient information and avoids the “vanishing gradient” problem (10). Each convolution layer is followed by a Batch Normalization (11) and Leaky ReLU (12) activation. We perform downsampling in our convolution operation and upsampling with a transpose convolution operation. The very last convolution layer converts a 64 channel input to a 3-channel output image. We employ Adaptive stochastic gradient descent (Adam) (13) as our optimizer. The U-Net architecture was chosen due to its lower complexity and data requirements than recently used adversarial networks that might overfit the training dataset, since the data sufficiency problem has not been addressed in deep learning based synthetic CT literature.

Of the 60 patients, 47 were used for training. To increase the diversity of imaging features on similar anatomic cross sections, sagittal planes were used for training. A total of 9,192 images were used for training. We also implemented data augmentation by random rotation of image by 90 degrees and also by randomly cropping a section of each image for training. To explore the impact of magnitude (and by implication, diversity) of training data, subsets of 10, 20, and 50% of total images were also tested, and the resulting MRCT images from test subjects qualitatively reviewed.

### Loss Function

The choice of loss function (L) for our task was a combination of mean absolute error (MAE) and mean squared error (MSE) losses between the CT and MRCT images.

(1) $$\begin{array}{r} MAE\left(X,Y\right)=\;\frac{\sum_{i=1}^{n}|X_{i}-Y_{i}|}{n} \end{array}$$

(2) $$\begin{array}{r} MSE\left(X,Y\right)=\frac{\sum_{i=1}^{n}\left(X_{i}^{2}-Y_{i}^{2}\right)}{n} \end{array}$$

(3) $$\begin{array}{r} L\left(X,Y\right)=MAE\left(X,Y\right)+MSE\left(X,Y\right) \end{array}$$

where *X, Y* are the images being compared, *n* is the total number of pixels in the image and *X*_*i*_ represents the *ith* pixel for image *X*.

We compare the loss *L* for each region separately which is backprojected for training the network:

(4) $$\begin{array}{r} L_{tot}\left(X,Y\right)\;=\;L\left(X_{air},Y_{air}\right)\;+L\left(X_{tissue},Y_{tissue}\right)\;+\;L\left(X_{bone},Y_{bone}\right) \end{array}$$

### Network Training

**Figure 3:**
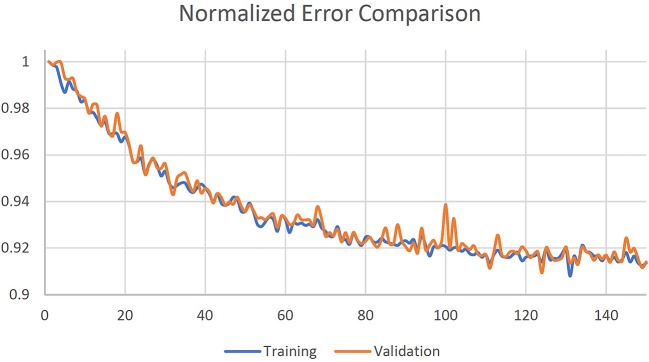
Normalized training and validation loss curves.

The U-net was initialized with a normal distribution with mean 0 and standard deviation 0.01. Training was done in mini-batches of 32 random slices. Five-fold cross validation was used, and training was stopped after 150 epochs where loss function was observed to reach a plateau as shown in Figure 3. The 3 channel images were summed along the channels dimension to generate corresponding MRCT slices.

### MRCT Evaluation

MRCT volumes were compared with corresponding CT volumes by various methods. MAE comparisons were done on voxel wise basis, as well as for voxels primarily containing air, soft tissue and bone. These regions were defined within an automatically generated mask that encompassed the head to the inferior border of the skull by using *dipy* (8).

Dosimetric comparisons were made on 11 targets from 7 of the test patients. Using a commercial treatment planning system (Eclipse, Varian Medical Systems, Palo Alto, CA), treatment plans for these radiosurgical targets were generated using the clinical treatment planning directives and with electron density maps derive using MRCT images. The beam fluences generated from these plans were used to recalculate doses by applying the aligned treatment planning CT image volumes as attenuation maps.

For these patients, the MRCT and treatment planning CT image volumes were individually aligned to the Cone Beam CT (CBCT) images acquired for treatment positioning. The alignment transformations were subsequently applied to the center of the planned treatment targets, and the differences in transformed coordinates compared.

## Results

The network training times were 928, 634, 302, and 161 min using 9192, 4096 (50%), 1838 (20%), and 919 (10%) image pairs, respectively, on 2 NVIDIA K40 GPUs. Generation of 3-channel MRCT images took ~1 s.

**Figure 4:**
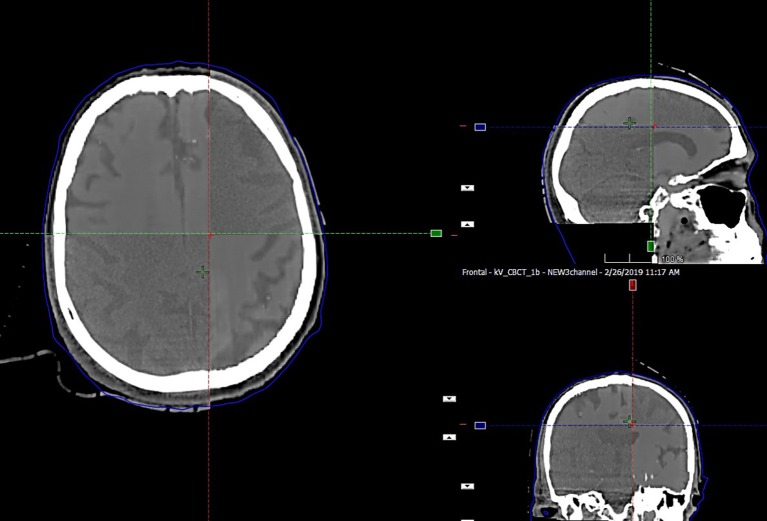
Split window display of MRCT aligned with CBCT for an example patient, demonstrating the preservation of dominant soft tissue interfaces such as major sulci and ventricles as seen in axial, coronal, and sagittal cross sections through the image volumes.

The preservation of major soft tissue interfaces is demonstrated in example images in Figure 4, which further shows support for soft tissue-based alignment between MRCT and CBCT. The MAE for the 13 test patients is reported in Table 1. The MAE for all voxels ranged from 58.1–118.1 HU with mean 81.0 HU and standard deviation 14.6 HU. Error values for each of the 3 channels are reported in Table 1. Mean MAE values for air, tissue and bone were 234, 22, and 193 HU, respectively.

**Table 1:** MAE values between MRCT and CT image volumes from the fully sampled network.

**Patient**	**MAE (HU)** **all voxels**	**MAE (HU)** **air**	**MAE (HU)** **tissue**	**MAE (HU)** **bone**
Patient 1	73.80	213.5	13.97	170.02
Patient 2	76.88	227.54	14.39	182.83
Patient 3	88.31	234.27	17.11	217.24
Patient 4	77.53	229.34	17.87	174.08
Patient 5	99.28	271.21	17.8	227.25
Patient 6	69.17	220.9	15.11	169.53
Patient 7	118.14	293.7	26.5	302.54
Patient 8	83.20	218.69	23.51	188.98
Patient 9	58.13	197.96	15.56	154.99
Patient 10	71.45	216.57	17.15	181.51
Patient 11	75.66	200.88	16.7	158.48
Patient 12	89.04	274.47	16.97	214.17
Patient 13	72.66	239.77	16.29	169.13
Mean	81.02	233.75	17.61	193.13
Standard deviation	14.60	28.02	3.41	38.33
Minimum	58.13	197.96	13.97	154.99
Maximum	118.14	293.7	26.5	302.54

**Figure 5:**
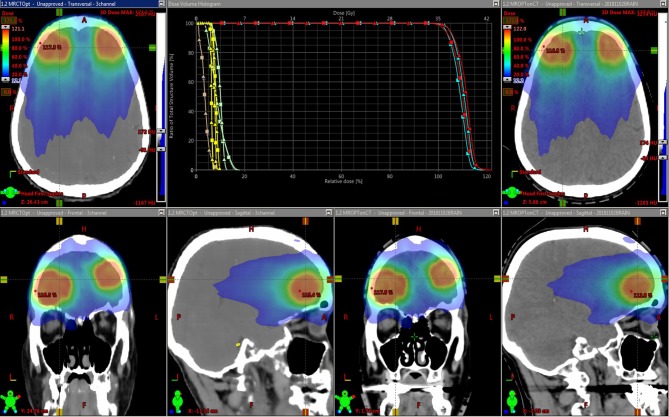
Dose distributions for intensity modulated treatment plans for two targets. The original plan was optimized using the MRCT-derived density grid (left), and the resulting beam fluences were used to recalculate doses on the CT-derived density grid (right). Dose volume histograms (DVHs) for the Brainstem (yellow), Optic chiasm (brown), eyes (green), and two targets (light and dark blue) are shown. Squares represent MRCT plan DVH curves, and triangles come from recalculated plans using CT.

Figure 5 shows an example treatment plan comparison. The PTV mean dose values had a systematic difference of 2.3% (σ 0.1%) between the plans generated using the MRCT-defined density grids and recalculated using the CT-defined grids. As can be seen on the images, the MRCT was not trained to reproduce the immobilization device present on the CT, and thus these differences are expected due to the added attenuation of the mask.

Alignment results from CT to CBCT as well as corresponding MRCT-CBCT alignment showed a mean difference of −0.1 (σ 0.2) mm, −0.1 (σ 0.3) mm, and −0.2 (σ 0.3) mm about the left-right, anterior-posterior and cranial-caudal axes, respectively. The range of differences was (−0.3, 0.4), (−0.4, 0.3), and (−0.7, 0.2) mm about the same axes.

**Figure 6:**
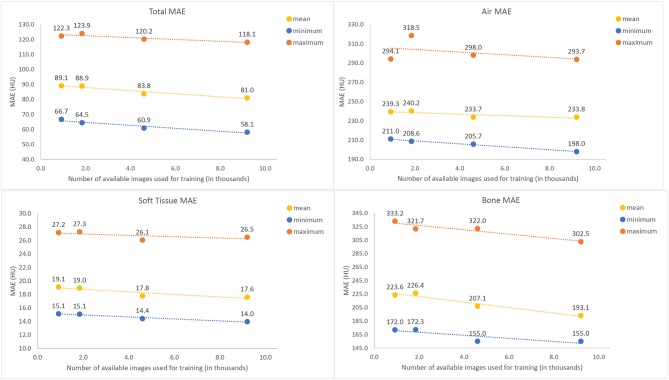
Mean Absolute Error (MAE) values between Synthetic and actual CT image volumes as a function of amount of training data used, tested across 13 tested patients. The maximum (orange), mean (purple), and minimum (blue) values all demonstrate a gradual decreasing trend with increased numbers of training images.

Figure 6 shows error for training the U-net with subsampled data for air, tissue and bone, respectively. While the difference between training on 10% (912) and 20% (1824) of available images is not clearly discernable, increasing the number of training images beyond 20% of the total 9192 available samples for training yielded a gradual decrease in average MAE for all three classes intensities, with the most significant trend observed in bony tissues. No plateau was observed, indicating that potential further improvements might be possible with a larger base set of training images.

## Discussion

In this report, we suggest an update to the design of neural networks used for generating synthetic CT from MRI. The goal of a 3-channel network is to allow learning of subtle contrast changes in HU values that might not be accurately learned due to the vast range of intensities in CT images. We implemented the 3-channel structure in a U-Net architecture and saw that soft-tissue contrast can be learned with good precision.

Two previous investigations reported MAE differences between synthetic and actual CT images within soft tissue regions. Emami (4) reported a MAE of 41.85 +/– 8.58 HU in soft tissue using a GAN trained on 15 patients, and Dinkla (6) reported a MAE of 22 +/– 3 HU using a dilated convolutional neural network trained on 52 patients. While we observed error values that are comparable or better (at least in soft tissue) than those reported in these and other investigations (1, 14), we would nonetheless argue that low MAE values are not enough for clinical implementation of MRI-only radiotherapy. Alignment of CT and CBCT is a crucial step that requires correct soft-tissue contrast, and a 3-channel network optimizes for it. We show that the 3-channel output network potentially reduces the problem of faithfully preserving soft tissue features by separately training on CT images within an appropriate intensity range. This process also allows us to scale the loss function to incur heavier penalties separately for errors for each of the different intensity regions.

We observed a gradual trend toward decreasing MAE with increasing amounts of training data. Many prior investigations used far fewer patient images for training than the 47 we had available, and it may be possible that their results are potentially limited by the amount of data available. It is likely that our results are limited by the amount of available data as well, and future investigations will focus on increasing the training data set to incorporate ideally hundreds of patients. A critical question for future investigations will be the elucidation of the necessary complexity of training information and robust estimation of resulting uncertainty from trained networks. While we chose to focus on a U-Net for training our data in part to limit the potential overfitting due to degeneracy associated with optimizing a network with a larger number of degrees of freedom from limited data, it is also possible that use of a generative adversarial network (GAN) may better reveal the relationship between volume and by inference complexity of training data and accuracy of final results. We will explore the use of GANs as we increase our training data in the future. Of note, a recently published study using a GAN trained on 77 patients with mutual information as a loss function reported an average MAE of 47.2, compared to a MAE of 60.2 when MAE was used as the loss function using the same network (14). While we combined L1 (MAE) and L2 (MSE) in our loss function, we clearly see the value in evaluating loss functions that are better designed to preserve local features, and will consider optimizing such functions in future investigations.

While we chose to train on 2-dimensional images in this investigation, other investigators have shown interesting results using “2.5 dimensional” groupings of multiple images in the same or orthogonal orientations, as well as through training on several 3-dimensional patches. These techniques, as well as nominally fully three-dimensional training, will be part of our future focus.

## Conclusion

A deep learning approach, consisting of simultaneous training of conversion of T1-weighted MR images to 3 separate intensity regions of corresponding spatially aligned CT images representing HU values typically found in voxels containing mostly air, soft tissue and bone, respectively, was investigated. Results indicate potential promise in preserving local soft tissue features. Furthermore, the potential advantage of increasing the volume of training data indicated potential further improvements with additional number of patients.

## Data Availability Statement

The datasets generated for this study are available on request to the corresponding author.

## Ethics Statement

The studies involving human participants were reviewed and approved by University of Michigan IRB. The patients/participants provided their written informed consent to participate in this study.

## Author Contributions

DG designed the study, conceived of the network design, collated the training data, and performed all training and related MAE analyses. MK accrued patients to the protocol for analyses, and provided the clinical treatment planning directives used for dose comparisons. KV performed the treatment planning, dose calculations and comparisons, and alignments with cone beam CT images. JB conceived the investigation, oversaw all aspects of the study design and execution, and collated analyses of data. DG and JB wrote the manuscript.

### Conflict of Interest

The authors declare that the research was conducted in the absence of any commercial or financial relationships that could be construed as a potential conflict of interest.
